# Anti-IgE therapy for IgE-mediated allergic diseases: from neutralizing IgE antibodies to eliminating IgE^+^ B cells

**DOI:** 10.1186/s13601-018-0213-z

**Published:** 2018-07-18

**Authors:** Jiayun Hu, Jiajie Chen, Lanlan Ye, Zelang Cai, Jinlu Sun, Kunmei Ji

**Affiliations:** 1 Department of Biochemistry and Molecular Biology, School of Medicine of Shenzhen University, Shenzhen, 518035 China; 2 Department of Allergy, Peking Union Medical College Hospital, Peking Union Medical College and Chinese Academy of Medical Sciences, Beijing, 100730 China

**Keywords:** Anti-IgE therapy, Allergic diseases, IgE^+^ B cells, IgE neutralization

## Abstract

Allergic diseases are inflammatory disorders that involve many types of cells and factors, including allergens, immunoglobulin (Ig)E, mast cells, basophils, cytokines and soluble mediators. Among them, IgE plays a vital role in the development of acute allergic reactions and chronic inflammatory allergic diseases, making its control particularly important in the treatment of IgE-mediated allergic diseases. This review provides an overview of the current state of IgE targeted therapy development, focusing on three areas of translational research: IgE neutralization in blood; IgE-effector cell elimination; and IgE^+^ B cell reduction. IgE-targeted medicines such as FDA approved drug Xolair (Omalizumab) represent a promising avenue for treating IgE-mediated allergic diseases given the pernicious role of IgE in disease progression. Additionally, targeted therapy for IgE-mediated allergic diseases may be advanced through cellular treatments, including the modification of effector cells.

## Background

The incidence of allergic diseases has been rising with increasing industrialization and the accompanying changes to the environment and people’s lifestyles. According to a 30-nation/region epidemiological investigation conducted by the World Allergy Organization (WAO) Specialty and Training Council, approximately 250 million (22%) of the 1.2 billion people in those regions suffered from allergic diseases, such as allergic rhinitis, allergic asthma, allergic conjunctivitis, eczema, food allergies and drug allergies etc. [1]. Meanwhile, according to the World Health Organization (WHO), the international incidence of asthma specifically has increased from 150 million people in 2005 to 300 million by 2012 [2]. In their White Book on Allergy, the WAO projects the asthma incidence number to reach 400 million by 2025 and estimates that asthma is lethal in about 250 thousand patients per year [3]. Because of their high prevalence and high recurrence rate, allergic diseases pose a serious financial burden for affected households and consume substantial resources in socialized healthcare systems. Thus, the WHO has designated allergic diseases as one of the top disease classes requiring major research and prevention measures in the 21st century [4].

## IgE is a key molecular in the development of IgE-mediated allergic diseases

The mechanism of allergic diseases is complex. In clinical practice, allergic diseases could be divided into two categories: IgE mediated and non-IgE mediated. Allergic disorders are characterized by inflammatory responses involving many types of cells, including mast cells and basophils, and various biological molecules, including cytokines (e.g. interleukin 4) and soluble mediators (e.g. histamine) [5]. IgE, an antibody class found only in mammals, has unique properties and plays a central role in IgE-mediated allergic diseases. Typically, it is the least abundant Ig isotype, with a concentration of about 150 ng/ml, compared with 10 mg/ml for IgG in the circulation of healthy individuals [6]. The half-life of IgE in serum is about 3 days, compared with 20 days for IgG, but its lifespan can be extended to 2 weeks in skin tissue [6]. Specific IgEs are upregulated in response to exposure to specific allergens. There are two types of IgE molecules: free IgE produced by plasma cells and membrane-bound IgE maintained on the surface membranes of B cells through class switching [7].

### IgE structure and properties

IgE shares the same basic molecular structure of other Igs, with two identical heavy chains and two identical light chains. However, the heavy ε-chain of IgE contains one more domain than the heavy γ-chain of IgG. The Cε3 and Cε4 domains of IgE are sequence homologous and structurally similar to the Cγ2 and Cγ3 domains of IgG; two Cε2 domains, which are the most obvious distinguishing feature of IgE, are present in place of the flexible hinge region found in IgG [8–10]. The Cε2 domains fold back and make contact with the Cε3 and Cε4 domains, perhaps acting as a spacer region between the Fab (antigen-binding fragment) arms and Fc (crystallizable fragment) region, and the compact, bent Cε2 domains provide considerable flexibility in the conformation of IgE [7].

IgE is a glycoprotein with a molecular mass of 190 KD and a sedimentation coefficient of 8S [11]. It is non-thermostable, losing its binding ability after 4 h at 56 °C [12]. The normal range of serum IgE levels is 50–300 ng/mL, markedly lower than typical IgG levels (~ 10 mg/ml) [13]. Serum IgE concentration can fluctuate dramatically. Patients with an allergic disease may have tenfold higher than normal serum IgE levels, and patients with a parasitic or fungal infection can be 1000-fold higher than normal [14]. The half-life of free IgE in the blood is short of ~ 3 days, which makes neutralizing therapy challenging. However, cell surface-bound IgE on basophils, mast cells, and dendritic cells can extend their activity to several weeks, or even several months [6]. When divalent or polyvalent antigens bind cell-surface IgE, they can induce the release of intracellular bioactive substances in the presence of Ca^2+^, thereby triggering an allergic reaction [15].

### IgE plays a critical role in the development of allergic diseases

IgE plays a key role in mediating the initiation and development of allergic diseases. The IgE molecule performs its biological function by binding its receptors on target cells, activating the induction immunomodulatory and protecting against parasitic worms (helminths) and the expulsion of environmental substances that include toxins, venoms, irritants and xenobiotics [12]. When the body encounters an allergen for the first time, IgM molecules in B cells are converted by class switching to IgE molecules [5]. Then, specific B cells initiate an IgE response, during which IgE^+^ B cells start to secrete free IgE into the blood where it can bind the high-affinity IgE receptor FcεR I on mast cells or basophils [16]. This response sensitizes the organism so that when itre-encounters the allergen, the allergen molecules are bound by specific IgE molecules on the surface of mast cells, causing them to undergo degranulation and to synthesize and release large quantities of allergic mediators (i.e., histamine, leukotriene, and platelet-activating factor), which then produce a local or systemic allergic reaction [17]. Apart from binding FcεRI on mast cells and basophils, IgE can also bind its low-affinity receptor FcεRII, which is expressed by B cells and monocytes [18]. Surface receptors on B cells enable them to take in bound allergens, process them, and present them to T cells, priming subsequent targeted innate immunity responses to the allergen in the future [19]. Anti-IgE therapy can reduce IgE receptor expression on effector cells; because IgE is a positive regulator of both FcεRI and FcεRII, reduced IgE results in reduced IgE receptor expression as well [20, 21]. With co-stimulation of CD79A (Ig-α) and CD79B (Ig-β), membrane-anchored IgE can also trigger the proliferation and differentiation of B cells [22].

## IgE targeted therapy for allergic diseases

There is great interest in the development of new drugs or methods that would alleviate allergic diseases by affecting molecular IgE activities in a manner that is safe, effective, and convenient (Table 1).

**Table 1:** Therapies to treat IgE-mediated allergic diseases

	Therapy	Type	Mechanism	Phase	References
Serum IgEneutralizization	Immuno adsorption	Plasma-pheresis	Removal of Ig and immune complex from blood	Marketed	Schmidt [23]
	Omalizumab	Monoclonal antibody	Bind Cε3 domain in heavy chain Fc of free IgE	Marketed	Chang [32]
	CMAB007	Biosimilar of omalizumab	Bind Cε3 domain in heavy chain Fc of free IgE	Phase III Local license	Bo Zhou [37]
	Ligelizumab	Monoclonal antibody	Target Cε3 domain of IgE	Phase II NCT01703312	Gauvreau [38]
	MEDI4212	Monoclonal antibody	Bind Cε3/Cε4 domain of IgE	Phase I NCT01544348	Sheldon [41]
	Recombinant ScFv	Single-chain antibody	Identify IgE, IgE-bound cells, and IgE-secreting cells	Preclinical	Lupinek [43]
IgE-effector cells	Anti-FcεRI Fab-conjugated celastrol-loaded micelles	Polymer	Prevent IgE interaction with mast cells, and kill mast cells	Preclinical	Peng [48]
	CTLA4Fcε	Fusion protein	Bind IgE receptors, FcεRI and FcεRII/CD23	Preclinical	Perez-Witzke [51]
IgE + B cells	Quilizumab	Monoclonal antibody	Target CεmX of IgE^+^ B cells	Phase II NCT01582503	Harris [59]
	Bsc-IgE/CD3	Monoclonal antibody	Eliminate IgE^+^ target cells by redirected T cells	Preclinical	Talay [61]
	XmAb7195	Monoclonal antibody	Form complex with B cell IgE receptors and FcγRII β	Phase I NCT02148744	Chu [66]

### Methods for neutralizing IgE in blood

**Fig. 1:**
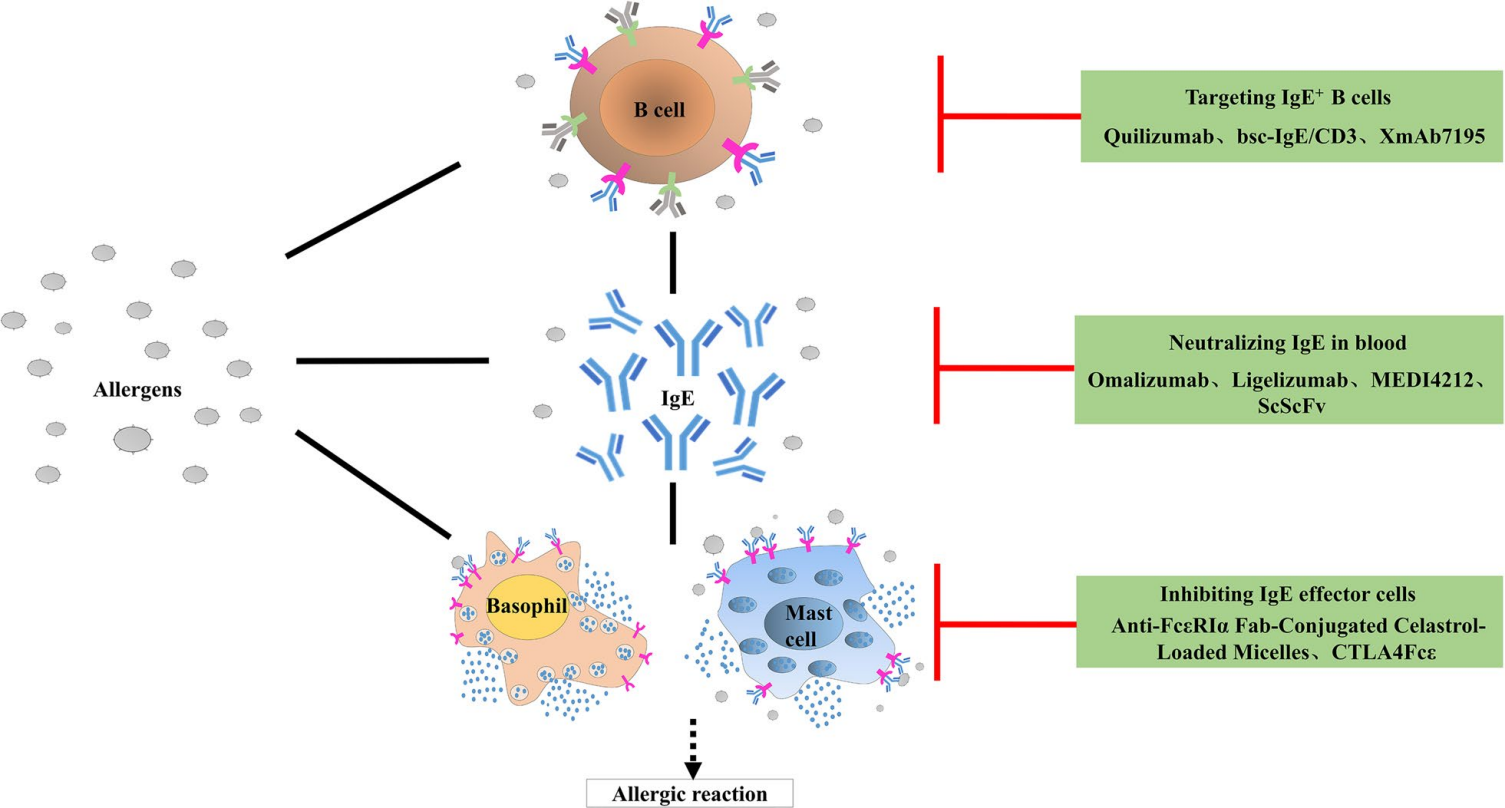
Scheme of anti-IgE therapy strategies for IgE-mediated allergic diseases. Through immunoadsorption, free IgEs in serum can be bound specifically and neutralized, thereby preventing IgE association with IgE receptors on target cells and thus suppressing early/late allergy reactions. CTLA4Fcε, and similar agents, suppress the emergence of allergic reactions by reducing the number of effector cells, and hence the quantity of allergic mediators. Immunological drugs, like quilizumab, alleviate allergies by suppressing IgE^+^ B cells and controlling IgE generation

IgE is an important target for allergic disease therapy. The main method of IgE neutralization being pursued involves achieving specific binding and neutralization of free IgE in serum to prevent it from binding receptors on target cells, thereby inhibiting allergen-induced early/late allergic reactions (Fig. 1).

#### Immunoadsorption

Immunoadsorption (IA), also called immune apheresis, has been adopted as an effective treatment for autoantibody-mediated diseases [23]. IA utilizes plasmapheresis to remove immunoglobulin and immune complexes and in cytapheresis, immune cells from the circulation [23]. Accordingly, IA could be successfully applied in patients with severe atopic dermatitis and high total serum IgE levels [24–27]. An IgE-specific adsorber, called IgEnio, has been developed [28]. The pilot study indicates that IgEnio may be used to treat pollen-induce allergic asthma [28].

#### Omalizumab

Omalizumab, developed by Novartis^®^, is a recombinant humanized monoclonal antibody against IgE [29]. It binds selectively to the Cε3 domain of the Fc fragment on the heavy chain of free IgE, reducing IgE availability significantly [30]. Because the Cε3 domain mediates IgE binding with the α chain of IgE receptors, omalizumab interferes with IgE-FcεRI interaction, thereby preventing mast cell/basophil degranulation and, ultimately, reducing the activation of inflammatory cells and the release of pro-inflammatory factors [31]. In addition, IgE binding of FcεRII on B cells supports antigen capture and Th2 activation. Thus, omalizumab can block IgE-mediated antigen-presenting processes and inhibit Th2 amplification of inflammatory reactions [32].

However, omalizumab is associated with safety concerns. Because it cannot reduce IgE levels quickly, it requires a long (several weeks), continuous treatment cycle [33]. The US Food and Drug Administration (FDA) warns that long-term use of omalizumab increases the risk of arterial thrombosis slightly and can have negative impacts on cardiac and cerebral circulation [34]. Because the long-term regime is costly, omalizumab is recommended primarily for severe asthma cases [35]. Besides, treatment with omalizumab for patients with chronic spontaneous urticaria resulted in clinical benefits after 12 weeks treatment since the levels of FcεRI and IgE expression on peripheral blood basophils were rapidly reduced [36].

In addition, CMAB007, a biosimilar of omalizumab, was developed by National Engineering Research Center of Antibody Medicine of China. It has the same amino acid sequence as omalizumab and have been finished phase III clinical trial in China approval by local departments [37]. Now, the drug CMAB007 for treating patients with allergic asthma is under large-scale clinical trials (NCT03468790) in China, which is a multi-centre, randomized, double-blind, placebo-controlled phase III study.

#### Ligelizumab (QGE031)

Ligelizumab, also developed by Novartis^®^, is a humanized IgG_1_ monoclonal antibodytargeting the Cε3 region of IgE [38]. Like omalizumab, ligelizumab inhibits the binding of free IgE to mast cells and basophils, thereby blocking the allergic reaction cascade and yielding clinical benefits to patients suffering from IgE-mediated allergic diseases.

Phase II clinical trials investigating the pharmacokinetics, pharmacodynamics, and safety of ligelizumab showed that it can decrease IgE levels more effectively than omalizumab via inhibition of IgE-FcεRI binding and that it can produce better outcomes, as indicated by skin prick allergen test responses [38, 39]. Hence, ligelizumab has the potential to be a good anti-IgE drug for allergy therapy.

#### MEDI4212

MEDI4212, a humanized IgG1λ monoclonal antibody generated by phage display technology, neutralizes free IgE by binding selectively to the Cε3 and Cε4 domains of IgE [40]. The affinity of MEDI4212 to human IgE was demonstrated to be 1.95 pM in vitro, which is one hundred times higher than that of omalizumab [40]. Because the Cε3 region is crucial for IgE interaction with its receptors, MEDI4212 inhibits the binding of IgE with FcεRI/FcεRII.

A phase I clinical trial (NCT01544348) showed that MEDI4212 is more effective than omalizumab in reducing serum IgE levels inpatients with IgE levels ≥ 30 IU/mL and that MEDI4212 treatment reduces FcεRI expression on dendritic cells and basophils [41]. However, pharmacokinetic analysis showed that MEDI4212 is removed rapidly in vivo, making long-term intake likely necessary for maintenance of IgE suppression [41].

The MEDI4121 may be mutagenized to improve the drug’s pharmacokinetic properties. A variant of MEDI4121 in which the Fc fragment was altered to enhance affinity for FcγRIIIa, which down-regulates IgE expression by B cells before they have differentiated into IgE secreting plasma cells [42]. Therefore, MEDI4121 variants are new immunotherapeutic candidates for both IgE neutralization and IgE^+^ B cell elimination.

#### Recombinant single-chain variable-fragment (ScFv) antibody

Recombinant ScFv is produced by cDNA encoding the heavy and light Ig chains. Biosensor-based studies have demonstrated that recombinant ScFv binds human IgE rapidly and efficiently (affinity, 1.52 × 10^−10^ M) [43]. It can bind cell-bound IgE (i.e., IgE^+^ B cells) as well as free IgE in vivo [43]. In vitro experiments showed that recombinant ScFv does not crosslink with IgE^+^ effector cells or trigger basophil/mast cell degranulation. Recombinant ScFv can also be used to probe IgE activities under both healthy and disease conditions, making it a helpful drug development tool.

### IgE effector cell inhibition

Immune effector cells, including mast cells, basophils, eosinophils, which are critical for the elimination of foreign substances and antigens, are the main sources pro-inflammatory factors. In particular, mast cells are the key cells that induce allergic asthma, and the number of mast cells in asthmatic patients is elevated significantly [44, 45]. After stimulation of allergens, mast cells secrete the autacoid mediators histamine, prostaglandin (PG) D2, and leukotriene (LT) C4, which are capable of inducing bronchoconstriction, mucus secretion, and mucosal edema, all features of asthma [44, 45]. Basophils degranulate for immediate release of histamine, rapidly generate LTC4, and produce Th2 cytokines provides the mechanistic basis whereby basophils can cause immediate hypersensitivity clinical symptoms [46]. The increased number of basophils are common during anaphylactic reaction [46]. Thus, reducing the number of effector cells reduces the material basis of anaphylaxis and can inhibit the fundamental development of allergic reactions.

#### Anti-FcεRI Fab-conjugated celastrol-loaded polymeric micelles

Celastrol is bioactive compound extracted from *Tripterygium wilfordii* (Thunder god vine) that can induce T cells apoptosis [47]. Thus, targeting celastrol specifically to mast cells in a manner that also reduces its toxicity is an attractive potential avenue for allergic disease treatment. This approach has been pursued by cross-linking celastrol with anti-FcεRI Fab, which has been shown to induce mast cell apoptosis, eliminating with them their pro-inflammatory factor cargo, and to limit celastrol toxicity [48]. Treatment of allergic asthma model mice with anti-FcεRIα Fab-conjugated polymeric micelles was shown to reduce secretion of inflammatory factors and eosinophil infiltration rapidly and to lead to remission of symptoms of ovalbumin-induced allergic inflammation symptoms [48]. The ability of anti-FcεRIα Fab-conjugated celastrol-loaded polymeric micelles to both block IgE binding of mast cells and induce mast cell apoptosis makes it a very attractive medicine for type I allergic diseases as well as for other mast cell-related diseases.

Anti-FcεRIα Fab-conjugated polymeric micelles have been shown to reduce allergic reactions more efficiently than omalizumab [48]. The following biochemical factors may underlie this favorable efficacy: (1) extension of pharmacokinetics by polymeric micelles; (2) promotion of drug aggregation in target tissues and target cells; and (3) competitive binding with FcεRI on the surface of mast cells resulting in reduced mast cell degranulation.

#### Synthetic cytotoxic T-lymphocyte-associated protein 4 (CTLA4) fused with Fcε

CTLA4 (a.k.a., CD152) is a protein receptor that, functioning as an immune checkpoint, down-regulates immune responses. It is constitutively expressed in CD4^+^CD25^+^ Foxp3^+^regulatory T cells, but is upregulated only in conventional T cellsupon activation. CTLA4 is homologous to the T cell co-stimulatory proteinCD28, and both molecules bind CD80(B7-1) and CD86(B7-2) on antigen-presenting cells [49]. It binds CD80 and CD86 with greater affinity and avidity than does CD28, thus enabling it to outcompete CD28 for its ligands [50]. CTLA4 transmits an inhibitory signal to T cells, whereas CD28 transmits a stimulatory signal [50].

Researchers have constructed a fusion protein containing the CD80/CD86-binding domain of CTLA-4 and the Fcε receptor-binding domain of the IgE H chain [51]. This recombinant protein binds both FcεRI/FcεRII and CTLA-4 receptors (i.e., CD80 and CD86), thereby suppressing Th2 responses. CTLA4 Fcɛ and CD23-CD80/CD86 combine to form a multi-molecule polymer, which acts as a spacer to influence production of soluble CD23. In an experiment involving human peripheral blood mononuclear cell samples stimulated in vitro, CTLA4 Fcɛ reduced the rate of lymphocyte proliferation in the presence of the lectin concanavalin A; in the same experiment, CTLA4 Fcɛ was also shown to bind IgE receptors on effector cells, thereby influencing soluble CD23 biosynthesis and inhibiting lymphocyte proliferation [51]. Given its demonstrated ability to affect IgE levels and the generation of IgE-secreting cells, the recombinant fusion protein CTLA4Fcɛ may be an effective medicine for controlling IgE-mediated immunodeficiency and other related diseases [51].

### Targeting IgE^+^ B cells

IgE^+^ B cells are critical for controlling IgE production. Both transient IgE secreted by plasma blasts in blood and long-living IgE secreted by plasma cells in bone marrow are influenced by IgE^+^ B cells [52].

#### Quilizumab (h47H4)

Membrane-bound IgE on the surface of B lymphocytes is of great importance for IgE production. It has an extra 52-amino acid-long CεmX-containing fragment between the CH4 domain of IgE and its B-cell membrane-anchoring segment [52, 53]. CεmX is the antigen-binding site of IgE-synthesis committed B cells [54, 55]. CεmX is both target-specific and cell-specific, making it a very suitable drug target.

Quilizumab, developed by Genentech^®^, is a new artificial monoclonal antibody that targets CεmX on IgE^+^ B cells. It produces crosslinking of membrane-bound IgE antigen receptors on B cells, which induces IgE^+^ B cell apoptosis, thereby reducing free IgE levels and inhibiting the generation of IgE^+^ B cells [56, 57]. Because the half-life of free IgE is quite short, this drug represents an efficient means with which to reduce IgE by eliminating the cells that express membrane IgE [58].

A phase II clinical trial showed that quilizumab is an effective candidate for treating allergic diseases safely and with high specificity [59]. Quilizumab was shown to lower total IgE and specific IgE levels in the serum of patients with asthma, and this effect lasted for 6 months [59]. It is hoped that quilizumab will be useful for the treatment and prevention of some IgE-mediated diseases, especially those for which there are no current medicines available [60]. However, quilizumab treatment did not produce a clinically meaningful benefit in allergic asthma patients inadequately controlled by standard therapy, despite its high ability in reducing serum IgE levels and the good tolerability profile [59].

#### Bispecific (bsc) IgE-CD3 antibody

There are several types of IgE^+^ B cells, including plasma blasts, plasma cells, and IgE^+^ memory B cells [61]. The earliest experiment aimed at eliminating IgE^+^ B cells specifically sought to modify T-cell receptors in combination with anti-IgE monoclonal antibody activity [62]. The bsc-IgE/CD3 antibody is an artificially modified targeting antibody specific for both IgE and CD3. It binds specifically to cells that express membrane-bound IgE and can re-direct the cytotoxicity of prestimulated human T cells toward IgE^+^ B cells, at least in vitro, without causing degranulation of mast cells or release of free IgE. Bsc-IgE/CD3 is an antibody that could eliminate both IgE^+^ B cells and free IgE in serum [63]. Therefore, bsc-IgE/CD3 is a new class candidate medicine for IgE-mediated allergic diseases.

#### XmAb7195

FcγRIIβ is involved in B-cell homeostasis and FcγRIIβ abnormalities lead to autoimmune diseases [64]. A novel antibody known as XmAb7195 was produced by humanization, affinity maturation, and Fc engineering using a murine anti-IgE antibody as template [65]. XmAb7195 can isolate free IgE in serum, forming immune complexes with FcγRIIβ and IgE receptors on B cells that impede the formation of IgE^+^ B cells and reduce free and total IgE levels without affecting the antigen isotypes of other B cells [66]. Because it has the added ability of binding FcγRIIβ, XmAb7195 can inhibit IgE^+^ B cell differentiation, thereby reducing the number of IgE secreting plasma cells [65]. This reliable double mechanism can be utilized to reduce total IgE levels, while the remaining free IgE can be targeted continuously and effectively. A phase I clinical trial (NCT02148744) showed that XmAb7195 is more efficient at reducing IgE activity than omalizumab [65].

## Prospective

As so far, Omalizumab is the only FDA-approved recombinant humanized monoclonal antibody that neutralizes IgE to treat allergic diseases [67]. Its use is supported by a large number of clinical trials demonstrating its effectiveness [68]. Other IgE-neutralizing antibodies are being developed with the goal of more effectiveness and less side effects. Medicines that target IgE effector cells and IgE^+^ B cells are also in development. Apart from targeting medicines, cellular treatments are also being developed. For example, T cells may be modified to bind anti-IgE T cell receptors, thereby reducing the number of IgE^+^ cells [62].

It is a great progress that another new drug dupilumab (Dupixent ^®^) was approved by the FDA in April 2017 for the treatment of adult patients with moderate-to-severe atopic dermatitis [69]. Dupilumab is a human monoclonal antibody targeting the interleukin-4 receptor (IL-4R) alpha subunit to block interleukin-4 (IL-4)/IL-13 signaling and to inhibit the inflammatory response that plays a role in the development of atopic dermatitis [70].

IgE-targeting is highly promising for the treatment of allergic diseases. Cellular treatments, including selective modification of effector cells, represent an innovative technology for targeted therapy of allergic diseases [71]. Notwithstanding, there remains a serious concern that reducing patients’ IgE response ability can increase their risk of cardiovascular disease [72]. Thus, evaluation of secondary effects of these medications in clinical settings remains vitally important.

## Conclusions

IgE is an important therapeutic target in IgE-mediated allergic diseases. Novel IgE targeted therapies are currently being developed, which focus on IgE neutralization in blood, IgE-effector cell elimination and IgE^+^ B cell reduction. More IgE-targeted medicines such as FDA approved drug Omalizumab are expected for treating IgE-mediated allergic diseases in the future.

## References

[1] Warner JO, Kaliner MA, Crisci CD, Del Giacco S, Frew AJ, Liu GH (2006). Allergy practice worldwide: a report by the World Allergy Organization Specialty and Training Council. Int Arch Allergy Immunol.

[2] Weinberg EG (2011). The WAO white book on allergy 2011-2012: review article. Curr Allergy Clin Immunol.

[3] Pawankar RCG, Holgate ST (2013). Wofld allergy organization (WAO) white book on allergy. Update.

[4] Nitin J, Palagani R, Shradha NH, Vaibhav J, Kowshik K, Manoharan R (2016). Prevalence, severity and risk factors of allergic disorders among people in south India. Afr Health Sci.

[5] Navines-Ferrer A, Serrano-Candelas E, Molina-Molina GJ, Martin M (2016). IgE-related chronic diseases and anti-IgE-based treatments. J Immunol Res.

[6] King CL, Poindexter RW, Ragunathan J, Fleisher TA, Ottesen EA, Nutman TB (1991). Frequency analysis of IgE-secreting B lymphocytes in persons with normal or elevated serum IgE levels. J Immunol.

[7] McCoy KD, Harris NL, Diener P, Hatak S, Odermatt B, Hangartner L (2006). Natural IgE production in the absence of MHC Class II cognate help. Immunity.

[8] Zheng Y, Shopes B, Holowka D, Baird B (1991). Conformations of IgE bound to its receptor Fc epsilon RI and in solution. Biochemistry.

[9] Zheng Y, Shopes B, Holowka D, Baird B (1992). Dynamic conformations compared for IgE and IgG1 in solution and bound to receptors. Biochemistry.

[10] Wan T, Beavil RL, Fabiane SM, Beavil AJ, Sohi MK, Keown M (2002). The crystal structure of IgE Fc reveals an asymmetrically bent conformation. Nat Immunol.

[11] Hnasko RM (2015). The biochemical properties of antibodies and their fragments. Methods Mol Biol.

[12] Sanjuan MA, Sagar D, Kolbeck R (2016). Role of IgE in autoimmunity. J Allergy Clin Immunol.

[13] Platts-Mills TA, Snajdr MJ, Ishizaka K, Frankland AW (1978). Measurement of IgE antibody by an antigen-binding assay: correlation with PK activity and IgG and IgA antibodies to allergens. J Immunol.

[14] Lawrence MG, Woodfolk JA, Schuyler AJ, Stillman LC, Chapman MD, Platts-Mills TA (2017). Half-life of IgE in serum and skin: consequences for anti-IgE therapy in patients with allergic disease. J Allergy Clin Immunol.

[15] Wurzburg BA, Tarchevskaya SS, Jardetzky TS (2006). Structural changes in the lectin domain of CD23, the low-affinity IgE receptor, upon calcium binding. Structure.

[16] Henault J, Riggs JM, Karnell JL, Liarski VM, Li J, Shirinian L (2016). Self-reactive IgE exacerbates interferon responses associated with autoimmunity. Nat Immunol.

[17] Bang LM, Plosker GL (2004). Spotlight on omalizumab in allergic asthma. BioDrugs.

[18] Uermosi C, Zabel F, Manolova V, Bauer M, Beerli RR, Senti G (2014). IgG-mediated down-regulation of IgE bound to mast cells: a potential novel mechanism of allergen-specific desensitization. Allergy.

[19] Greiner AN, Hellings PW, Rotiroti G, Scadding GK (2011). Allergic rhinitis. Lancet.

[20] MacGlashan DW, Bochner BS, Adelman DC, Jardieu PM, Togias A, McKenzie-White J (1997). Down-regulation of Fc(epsilon)RI expression on human basophils during in vivo treatment of atopic patients with anti-IgE antibody. J Immunol.

[21] Arock M, Le Goff L, Becherel PA, Dugas B, Debre P, Mossalayi MD (1994). Involvement of Fc epsilon RII/CD23 and l-arginine dependent pathway in IgE-mediated activation of human eosinophils. Biochem Biophys Res Commun.

[22] Davis RE, Ngo VN, Lenz G, Tolar P, Young RM, Romesser PB (2010). Chronic active B-cell-receptor signalling in diffuse large B-cell lymphoma. Nature.

[23] Schmidt E, Zillikens D (2010). Immunoadsorption in dermatology. Arch Dermatol Res.

[24] Meyersburg D, Schmidt E, Kasperkiewicz M, Zillikens D (2012). Immunoadsorption in dermatology. Ther Apheresis Dial.

[25] Bresci G, Romano A, Mazzoni A, Scatena F, Altomare E, Capria A (2010). Feasibility and safety of granulocytapheresis in Crohn’s disease: a prospective cohort study. Gastroenterol Clin Biol.

[26] Soerensen H, Schneidewind-Mueller JM, Lange D, Kashiwagi N, Franz M, Yokoyama T (2006). Pilot clinical study of Adacolumn cytapheresis in patients with systemic lupus erythematosus. Rheumatol Int.

[27] Sakai Y, Sakai S, Otsuka T, Ohno D, Murasawa T, Munakata K (2009). Efficacy of high-throughput leukocytapheresis for rheumatoid arthritis with a reduced response to infliximab. Ther Apheresis Dial.

[28] Lupinek C, Derfler K, Lee S, Prikoszovich T, Movadat O, Wollmann E (2017). Extracorporeal IgE immunoadsorption in allergic asthma: safety and efficacy. EBioMedicine.

[29] Presta LG, Lahr SJ, Shields RL, Porter JP, Gorman CM, Fendly BM (1993). Humanization of an antibody directed against IgE. J Immunol.

[30] Zheng L, Li B, Qian W, Zhao L, Cao Z, Shi S (2008). Fine epitope mapping of humanized anti-IgE monoclonal antibody omalizumab. Biochem Biophys Res Commun.

[31] Eggel A, Baravalle G, Hobi G, Kim B, Buschor P, Forrer P (2014). Accelerated dissociation of IgE-FcepsilonRI complexes by disruptive inhibitors actively desensitizes allergic effector cells. J Allergy Clin Immunol.

[32] Chang TW, Wu PC, Hsu CL, Hung AF (2007). Anti-IgE antibodies for the treatment of IgE-mediated allergic diseases. Adv Immunol.

[33] Holgate SBJ, Wenzel S (2001). Efficacy of omalizumab, all anti-immunoglobulin E antibody, in patients with allergic asthma at high risk of serious asthma-related morbidity and mortality. Curt Med Res Opin.

[34] USFaDA. FDA Drug Safety Communication: FDAapproves label changes for asthma drug Xolair (omalizumab), including describing slightly higher risk of heart and brain adverse events. http://www.fda.gov/drugs/drugsafety/ucm414911.htm. Accessed Sept 29, 2014.

[35] Holgate STCA, Hebeft J (2004). Efficacy and safety of a recombinant anti-immunoglobulin E antibody(omalizumab)in severe allergic asthma. Clin Exp Allergy J Br Soc Allergy Clin Immunol.

[36] Metz M, Staubach P, Bauer A, Brehler R, Gericke J, Kangas M (2017). Clinical efficacy of omalizumab in chronic spontaneous urticaria is associated with a reduction of FcepsilonRI-positive cells in the skin. Theranostics.

[37] Zhou B, Lin B, Li J, Qian W, Hou S, Zhang D (2012). Tolerability, pharmacokinetics and pharmacodynamics of CMAB007, a humanized anti-immunoglobulin E monoclonal antibody, in healthy Chinese subjects. mAbs.

[38] Gauvreau GM, Arm JP, Boulet LP, Leigh R, Cockcroft DW, Davis BE (2016). Efficacy and safety of multiple doses of QGE031 (ligelizumab) versus omalizumab and placebo in inhibiting allergen-induced early asthmatic responses. J Allergy Clin Immunol.

[39] Arm JP, Bottoli I, Skerjanec A, Floch D, Groenewegen A, Maahs S (2014). Pharmacokinetics, pharmacodynamics and safety of QGE031 (ligelizumab), a novel high-affinity anti-IgE antibody, in atopic subjects. Clin Exp Allergy J Br Soc Allergy Clin Immunol.

[40] Cohen ES, Dobson CL, Kack H, Wang B, Sims DA, Lloyd CO (2014). A novel IgE-neutralizing antibody for the treatment of severe uncontrolled asthma. mAbs.

[41] Sheldon E, Schwickart M, Li J, Kim K, Crouch S, Parveen S (2016). Pharmacokinetics, pharmacodynamics, and safety of MEDI4212, an anti-IgE monoclonal antibody, in subjects with atopy: a phase I study. Adv Therapy.

[42] Nyborg AC, Zacco A, Ettinger R, Jack Borrok M, Zhu J, Martin T (2016). Development of an antibody that neutralizes soluble IgE and eliminates IgE expressing B cells. Cell Mol Immunol.

[43] Lupinek C, Roux KH, Laffer S, Rauter I, Reginald K, Kneidinger M (2009). Trimolecular complex formation of IgE, Fc epsilon RI, and a recombinant nonanaphylactic single-chain antibody fragment with high affinity for IgE. J Immunol.

[44] Brown JM, Wilson TM, Metcalfe DD (2008). The mast cell and allergic diseases: role in pathogenesis and implications for therapy. Clin Exp Allergy J Br Soc Allergy Clin Immunol.

[45] Bradding P, Walls AF, Holgate ST (2006). The role of the mast cell in the pathophysiology of asthma. J Allergy Clin Immunol.

[46] Cromheecke JL, Nguyen KT, Huston DP (2014). Emerging role of human basophil biology in health and disease. Curr Allergy Asthma Rep.

[47] Sethi G, Ahn KS, Pandey MK, Aggarwal BB (2007). Celastrol, a novel triterpene, potentiates TNF-induced apoptosis and suppresses invasion of tumor cells by inhibiting NF-kappaB-regulated gene products and TAK1-mediated NF-kappaB activation. Blood.

[48] Peng X, Wang J, Li X, Lin L, Xie G, Cui Z (2015). Targeting mast cells and basophils with anti-FcεRIα Fab-conjugated celastrol-loaded micelles suppresses allergic inflammatio. J Biomed Nanotechnol.

[49] Takahashi T, Tagami T, Yamazaki S, Uede T, Shimizu J, Sakaguchi N (2000). Immunologic self-tolerance maintained by CD25(+)CD4(+) regulatory T cells constitutively expressing cytotoxic T lymphocyte-associated antigen 4. J Exp Med.

[50] Krummel MF, Allison JP (1995). CD28 and CTLA-4 have opposing effects on the response of T cells to stimulation. J Exp Med.

[51] Perez-Witzke D, Miranda-Garcia MA, Suarez N, Becerra R, Duque K, Porras V (2016). CTLA4Fcepsilon, a novel soluble fusion protein that binds B7 molecules and the IgE receptors, and reduces human in vitro soluble CD23 production and lymphocyte proliferation. Immunology.

[52] Chen JB, Wu PC, Hung AF, Chu CY, Tsai TF, Yu HM (2010). Unique epitopes on C epsilon mX in IgE-B cell receptors are potentially applicable for targeting IgE-committed B cells. J Immunol.

[53] Chen HY, Liu FT, Hou CM, Huang JS, Sharma BB, Chang TW (2002). Monoclonal antibodies against the C(epsilon)mX domain of human membrane-bound IgE and their potential use for targeting IgE-expressing B cells. Int Arch Allergy Immunol.

[54] Batista FD, Anand S, Presani G, Efremov DG, Burrone OR (1996). The two membrane isoforms of human IgE assemble into functionally distinct B cell antigen receptors. J Exp Med.

[55] Peng C, Davis FM, Sun LK, Liou RS, Kim YW, Chang TW (1992). A new isoform of human membrane-bound Ige. J Immunol.

[56] Gauvreau GM, Harris JM, Boulet LP, Scheerens H, Fitzgerald JM, Putnam WS (2014). Targeting membrane-expressed IgE B cell receptor with an antibody to the M1 prime epitope reduces IgE production. Sci Transl Med.

[57] Scheerens H, Zheng Y, Wang Y, Mosesova S, Maciuca R, Liao XC, Wu LC, Matthews JG, Harris JM (2012). Treatment with Memp 1972a, an anti-M1 prime monoclonal antibody, reduced serum Ige in healthy volunteers and patients with allergic rhinitis. Am J Respir Crit Care Med.

[58] Brightbill HD, Jeet S, Lin Z, Yan D, Zhou M (2010). Antibodies specific for a segment of human membrane IgE deplete IgE-producing B cells in humanized mice. J Clin Investig.

[59] Harris JM, Maciuca R, Bradley MS, Cabanski CR, Scheerens H, Lim J (2016). A randomized trial of the efficacy and safety of quilizumab in adults with inadequately controlled allergic asthma. Respir Res.

[60] Liour SS, Tom A, Chan YH, Chang TW (2016). Treating IgE-mediated diseases via targeting IgE-expressing B cells using an anti-CepsilonmX antibody. Pediatr Allergy Immunol.

[61] Talay O, Yan DH, Brightbill HD, Straney EEM, Zhou MJ, Ladi E (2013). IgE(+) memory B cells and plasma cells generated through a germinal-center pathway. Nat Immunol.

[62] Lustgarten J, Eshhar Z (1995). Specific elimination of Ige production using T-cell lines expressing chimeric T-cell receptor genes. Eur J Immunol.

[63] Kirak ORG (2015). A novel, nonanaphylactogenic, bispecific IgE-CD3 antibody eliminates IgE(+) B cells. J Allergy Clin Immunol.

[64] Pritchard NR, Cutler AJ, Uribe S, Chadban SJ, Morley BJ, Smith KG (2000). Autoimmune-prone mice share a promoter haplotype associated with reduced expression and function of the Fc receptor FcgammaRII. Curr Biol.

[65] Chu SY, Horton HM, Pong E, Leung IW, Chen H, Nguyen DH (2012). Reduction of total IgE by targeted coengagement of IgE B-cell receptor and FcgammaRIIb with Fc-engineered antibody. J Allergy Clin Immunol.

[66] Chu SY, Yeter K, Kotha R, Pong E, Miranda Y, Phung S (2014). Suppression of rheumatoid arthritis B cells by XmAb5871, an anti-CD19 antibody that coengages B cell antigen receptor complex and Fcgamma receptor IIb inhibitory receptor. Arthritis Rheumatol.

[67] Kawakami T, Blank U (2016). From IgE to omalizumab. J Immunol.

[68] Tonacci A, Billeci L, Pioggia G, Navarra M, Gangemi S (2017). Omalizumab for the treatment of chronic idiopathic urticaria: systematic review of the literature. Pharmacotherapy.

[69] Boozalis E, Semenov YR, Kwatra SG (2018). Food and drug administration approval process for dermatology drugs in the United States. J Dermatol Treat.

[70] Han Y, Chen Y, Liu X, Zhang J, Su H, Wen H (2017). Efficacy and safety of dupilumab for the treatment of adult atopic dermatitis: a meta-analysis of randomized clinical trials. J Allergy Clin Immunol.

[71] Kuo CY, Kohn DB (2016). Gene therapy for the treatment of primary immune deficiencies. Curr Allergy Asthma Rep.

[72] Magen E, Mishal J, Vardy D (2015). Selective IgE deficiency and cardiovascular diseases. Allergy Asthma Proc.

